# Case in Which Fatal Consequences Arose from an Injury to the Ankle, in Some Gymnastic Exercises

**Published:** 1827-10

**Authors:** Thomas Rose

**Affiliations:** Surgeon to St. George's Hospital.


(To be continued.)

---

### INJURY OF THE ANKLE.

*Case in which fatal Consequences arose from an Injury to the Ankle, in some Gymnastic Exercises.* By Thomas Rose, Esq. Surgeon to St. George's Hospital.

The importance of well-regulated exercise to the full development of the muscular powers is unquestionable, and great advantages may, no doubt, be expected to result from the attention which has of late been bestowed in securing its systematic employment in schools and public institutions. It is, however, to be apprehended that the gymnastic exercises which are now so generally practised may, in some constitutions, be productive of bad effects. Many individuals have, from their peculiar conformation, a tendency to hernia, and in these, violent exertions of all kinds are attended with risk. It should be recollected that the bones are slow in acquiring their full degree of firmness and strength,—in some constitutions particularly so; that the extremities of the long bones are not in early life united to the bodies, but in the state of epiphyses, which anchylôse, gradually, towards the period of puberty; that, in early life, these epiphyses are liable to be detached by any sudden wrench or violence; and that the ligaments and cartilages by which they are connected with the shafts of the bones, may be seriously injured if brought to sustain any great degree of pressure or weight in particular positions of the body. Of the ill effects of inattention to some of these circumstances, the following case affords a melancholy example.

A young lady, twelve years of age, of a florid complexion, and who had always enjoyed good health, was sent from the north of England to a small boarding-school at Kensington, for the purposes of education. She received a lesson in dancing and gymnastics, from a distinguished teacher of these accomplishments, on the 28th of February in the present year. In that lesson, she had been made to rest the weight of her body repeatedly, and for several seconds at a time, first on the ball of one foot, and then of the other, and to stand in various positions, with a view of bringing the different muscles into action. The exertions, which these exercises required, were described, by a lady who was present, as having been very considerable and fatiguing. She was not aware of having sustained any particular wrench at the time, though, immediately after the lesson, she felt a good deal both of stiffness and of aching in her right ankle. She slept well that night, and she took, on each of the two succeeding days, (viz. on the 1st and 2d of March,) her usual walk in the garden. On both of them, however, she felt lame on the right ankle, and, from this being observed, she was directed to lie down on the rug during her lessons; a direction with which she appeared very ready to comply.

On the night of the 1st of March, after getting into bed, her ankle was so uneasy that she requested the maid, who attended on her and one or two of her companions, to rub it, which was done for some time before she could go to sleep. She kept entirely to a recumbent posture on the 3d of March, but, her disposition not leading her to complain, the lameness was supposed to proceed from chilblains, with which she was affected, and was not particularly attended to. A medical friend of her family was requested to see her on the 4th, and, in consequence of his report, the lady in whose care she was, brought her immediately to my house, where she arrived at six in the evening, requiring to be carried both to and from the carriage.

I found a considerable degree of puffiness and swelling over all her right ankle, but especially over the lower part of the tibia, with fluid thrown out amongst the ligaments and tendons, so that the right ankle was about an inch and a half in circumference more than the other. The foot and toes were pointed downwards, by a rigid contraction of the muscles on the back of the leg; and when, after some persuasion, she allowed me gently to bend her foot upwards, a convulsive action of the fibres of the gastrocnemius and solæus muscles was excited, so that the foot was kept in a state of continual tremor for two or three minutes. This was attended with but little pain, and, though there was a considerable degree of tenderness when pressure was made on the different parts about the ankle and instep, there was not, even on pressure, any thing like severe pain.

With these local symptoms there was a great deal of constitutional disturbance: her tongue was covered with a thick, white, mucous fur; her pulse was extremely quick and small; she complained of thirst; and her whole appearance exhibited the highest state of nervous irritability.

I ordered twelve leeches to be applied to the ankle, and, when the bleeding had ceased, that the parts should be covered with an evaporating lotion; and directed that she should take, as soon as she got into bed, a pill composed of five grains of Calomel and three of Extract of Poppy; an effervescing draught, with a scruple of Sulphate of Potash, every four hours; and a Senna draught early on the following morning; her diet being confined to tea and other diluents.

March 5th.—I visited her at Kensington in the evening. The swelling over the inner ankle, extending some way up the leg, had much increased; the skin exhibited a shining erysipelatous redness, especially round the leech-bites, which had bled freely. The pain in moving the foot had become very considerable.

She had passed an extremely restless night, her pulse continuing very quick, but less easily compressed than on the preceding evening; her tongue still white, and her skin hot and dry. The medicine had acted on her bowels; the motions were not copious, but very offensive.

I took away sixteen ounces of blood from her arm, which produced faintness; and directed four grains of Calomel to be given immediately, and a Senna draught, with some Tincture of Jalap, to be repeated in the morning; continuing the effervescing draughts every four hours, as well as the evaporating lotion; and laying the leg carefully on the outer ankle upon a pillow.

6th.—The tumefaction of the limb had extended up to the knee, the shining redness continuing about the inner ankle, and some way up the leg in the course of the tibia. The redness terminated towards the calf of the leg, that part being much swoln and tense, but the integuments there appearing more white than natural. She had been delirious in the night; her pulse was 130; her tongue continued white; she sighed frequently, had a hot skin, and great thirst. Her bowels had been freely opened. The blood taken last night was much cupped, and with a thick coat of buff.

I repeated the bleeding from her arm to the extent of eight ounces, which again induced faintness; and ordered a saline draught, with ten minims of Tincture of Digitalis, and twenty of Antimonial Wine, to be given every four hours.

In the evening, Mr. Wardrop saw her with me, and continued to do so for the next five or six days. The tumefaction had increased, but the redness had somewhat diminished. The other symptoms continued as in the morning. The blood last taken was very buffy. We agreed to repeat the bleeding to the extent of eight ounces, and to continue the evaporating lotion. At Mr. Wardrop's suggestion, the following medicine was prescribed:

℞. Vini Colchici m. x.; Magnesiæ Carbonatis ℈ss.; Aquæ Menthæ piper. ℥j. M. fiat haustus quartâ quâque horâ sumendus.

March 7th.—There was evidently fluid effused over the malleolus internus, extending some way up the tibia; her pulse was 144; the frequent sighing, and other symptoms of irritability, continued; and she had passed another very restless night.

The medicines, &c. to be continued as before.

8th.—Suppuration had evidently taken place to some extent, the quantity of fluid being increased. Her constitutional symptoms continued much the same. A free opening was made with an abscess-lancet a little above the inner ankle: several ounces of pus were discharged, and the parts were covered with a poultice.

She was directed to take a common saline draught, with a little Epsom salts, occasionally.

9th.—There had been a copious discharge of matter into the poultice; but a large collection was now perceptible on the fore part of the tibia, a little below the knee. I made, therefore, a very free incision with a scalpel, dividing the integuments to the extent of four or five inches, in the line of the tibia. The spine of the tibia, the whole of the fore part, and part of the outer surface of that bone, were felt to be denuded of the periosteum. Her pulse continued as high as 144, and her other constitutional symptoms were in no respect ameliorated.

To foment the limb, and continue poultices.—℞. Magnes. Sulphat. ʒj. Infusi Rosæ ℥jss. M. fiat haustus quater diê sumendus.

10th.—A copious discharge from both the openings, but the matter evidently penetrates every where between the bone and the periosteum. The two openings were therefore laid into one, by which the whole anterior surface of the tibia was laid bare, from the knee to the commencement of the malleolus internus. The periosteum was found to be separated from the bone in every direction, so that the bone lay as it were insulated, though still of a reddish tinge, and apparently perfectly healthy; bleeding slightly when touched with the sharp end of a probe. Her pulse, tongue, &c. as yesterday.

The same medicines were directed to be continued; to which, when I saw her in the evening, I added a composing draught.

11th.—There was no material change. The incision which had been made, terminated a little above the junction of the shaft of the tibia with the epiphysis, forming the inner ankle. A small membranous band over this part of the leg being divided to-day with the edge of a lancet, in consequence of matter lodging behind it, the epiphysis was found completely and entirely separated from the body of the tibia, and matter appeared oozing out between their extremities. It was at this part that the swelling first commenced, and it seems probable that the whole of the mischief arose either from the epiphysis being detached during the exercise which the young lady took on the 28th of February, or from some serious injury which was at that time done to the ligaments and cartilage by which that epiphysis was united to the lower portion of the tibia.

13th.—I placed a large splint along all the outer edge of the leg and foot, continuing the poultices, and supporting the limb carefully in every part. The pulse was reduced to 130. Very light nutriment has been allowed for several days past. She has had quieter nights, but opium disagrees.

15th.—The pulse is reduced to 120; she is much more tranquil. Granulations begin to arise from different parts of the bone; the limb is kept steadily supported, and the poultice is this day changed for a light dressing. She is allowed a little fish.

18th.—The epiphysis is obviously uniting itself to the shaft of the tibia, the bone at the extremity of each becoming very soft and vascular, and vessels shooting across from the one to the other; the two are dove-tailed together by numerous indentations. The limb is supported by straps of soap-cerate, with an eighteen-tailed bandage and the splint. Granulations have arisen more freely from the tibia, leaving only the fore part of it exposed, where the exfoliation does not appear likely to be very deep or extensive. The discharge is less. Unfortunately ulcers have formed both over the sacrum and over the trochanter of the right side, from her being so much confined to the same position, in spite of all the care that could be taken, by hollow pillows and other means, to guard against this calamity.

26th.—The arrival of her mother some days ago produced a considerable degree of excitement. She had previously been kept extremely quiet, and to this a very gentle disposition had materially contributed. The anxiety and distress, which the mother could not conceal, changed the appearances a good deal for the worse. She could not sleep on the night after her mother's arrival, had some degree of delirium, and an exacerbation of all the febrile symptoms. Since that her bowels have been a good deal disturbed, and her tongue and fauces are now covered with aphthæ. The limb continues quiet, and looks well. The ulcers on the sacrum and trochanter are painful.

29th.—The aphthous state of the fauces and tongue has increased, large films of lymph forming upon them. The bowels are very irritable, with some degree of tenderness over different parts of the abdomen, and considerable tension. She has taken castor-oil occasionally, with small doses of rhubarb in chalk mixture, and a few grains of Hydrargyrus cum Cretâ at night. The discharge from the leg has increased, and the granulations begin to separate more extensively from the bone.

It is not necessary to continue a further detail of these symptoms, which terminated fatally on the 3d of April.

*Park-place; Sept. 7th,* 1827.

